# Machine learning-based mortality risk prediction models in patients with sepsis-associated acute kidney injury: a systematic review

**DOI:** 10.3389/fmed.2025.1680180

**Published:** 2025-10-08

**Authors:** Xu Li, Xu Hu, Huiting Xu, Pin Yu, Hailing Ju

**Affiliations:** ^1^Department of Central ICU, The First Affiliated Hospital of Soochow University, Suzhou, China; ^2^School of Medicine, Tongji University, Shanghai, China; ^3^Department of Operating Room, Qingpu Branch of Zhongshan Hospital Affiliated to Fudan University, Shanghai, China; ^4^Department of Nursing, Shanghai Tenth People's Hospital, Shanghai, China

**Keywords:** machine learning, sepsis, acute kidney injury, mortality, predictive model, systematic review

## Abstract

**Background:**

Machine learning (ML) models are increasingly utilized to predict mortality in patients with sepsis-associated acute kidney injury (SA-AKI), frequently surpassing traditional scoring systems. Despite their efficacy, inconsistencies in model quality remain a concern. This review aims to evaluate existing ML-based SA-AKI mortality prediction models, with a focus on development quality, methodological rigor, and predictive performance.

**Objective:**

To systematically assess ML-based mortality risk prediction models for SA-AKI patients.

**Methods:**

A comprehensive literature search on ML-based SA-AKI mortality prediction models was conducted across PubMed, Cochrane, Embase, and Web of Science from the inception of these databases until July 2025. Two researchers independently screened the literature, extracted data, and assessed model quality employing the Prediction Model Risk of Bias Assessment Tool for Artificial Intelligence.

**Results:**

Nine studies were included, all of which entailed model development and validation phases; five were solely internally validated while four underwent external validation as well. The studies utilized 18 different algorithms, with Random Forest and Extreme Gradient Boosting being the most prevalent. The majority of the studies employed K-nearest neighbor or Multiple Imputation by Chained Equations for handling missing values and utilized Recursive Feature Elimination, Least Absolute Shrinkage and Selection Operator, and Boruta's algorithm for feature selection. Seven studies assessed model calibration performance. The Area Under the Curve (AUC) for the training sets generally ranged from 0.75 to 0.99, which decreased to 0.70 to 0.87 during internal validation. Extreme Gradient Boosting consistently showed robust performance in external validation. The final predictors encompassed six principal categories: demographic information, vital signs, laboratory tests, disease severity, comorbidities, and interventions.

**Conclusions:**

ML models demonstrate promising performance and applicability in predicting mortality risk in SA-AKI patients, with consistent core predictors. Nevertheless, most studies exhibit a potential risk of bias. Future efforts should aim to enhance the standardization of data processing, feature selection, and validation processes. Additionally, there is a need to focus on the construction of prospective models based on early variables, and to ensure the interpretability and clinical integration of the models to facilitate their practical application in healthcare workflows.

**Systematic review registration:**

identifier: CRD42025634551.

## 1 Introduction

Sepsis is characterized as an acute organ dysfunction syndrome precipitated by a dysregulated host immune response to infection, which exhibits substantially high morbidity and mortality rates (1). It is estimated that approximately 48.9 million individuals globally are afflicted by sepsis annually, resulting in about 11 million deaths. This accounts for nearly 20% of all global mortality figures (2). One of the most prevalent complications of sepsis, Acute Kidney Injury (AKI), markedly elevates the risk of mortality, extends hospital stays, and increases the necessity for renal replacement therapy in affected patients (3). Sepsis-associated Acute Kidney Injury (SA-AKI), a distinct phenotype of AKI, is often characterized by renal impairment that precedes clinical manifestations owing to its insidious onset and rapid progression. Systematic assessments indicate that the morbidity and mortality rates of SA-AKI range between 14% and 87%, and 11% and 77%, respectively (4). Furthermore, delays in mortality risk identification can critically impact the timing of interventions and prognoses; thus, the early identification of patients at high risk of mortality in SA-AKI and the development of personalized risk assessment tools represent crucial components of contemporary clinical critical care management.

Current clinical tools for assessing sepsis severity, such as the SOFA, APACHE II, and SAPS II scores, are commonly employed to evaluate organ function and overall mortality risk. However, these tools demonstrate poor generalization, possess limited predictive capabilities for the specific subgroup of SA-AKI, and exhibit low sensitivity and specificity. Additionally, they are typically one-time, static assessments that fail to dynamically reflect disease progression (5–7).

With advancements in artificial intelligence, Machine Learning (ML) algorithms have become increasingly prevalent in the medical field, particularly in the early identification of diseases (8–10), prognosis prediction (11–13), and clinical decision-making (14, 15). ML algorithms are capable of processing large-scale, multi-dimensional, non-linear, and highly interactive data sets. They can autonomously conduct feature selection and model optimization, thereby enabling real-time and dynamic predictions that can be seamlessly integrated into clinical information systems (16). Several scholars have employed ML models to predict mortality risk among SA-AKI patients in ICUs, with most reported predictive performance indicators surpassing those of traditional scoring systems. However, the reliability of these models remains questionable due to significant variances in data sources, variable processing, modeling algorithms, and model validation methods.

Consequently, this study aims to systematically review existing SA-AKI mortality prediction models that utilize ML algorithms, evaluate their development quality, predictive performance, clinical applicability, and risk of bias, and provide evidence-based recommendations to support the standardized application of AI models in ICU risk prediction.

## 2 Materials and methods

### 2.1 Literature inclusion and exclusion criteria

#### 2.1.1 Inclusion criteria

① Study participants aged ≥18 years. ② Patients diagnosed with sepsis. ③ Study designs encompassing cohort studies, case-control studies, and cross-sectional studies. ④ Models that predict mortality, employ ML to develop predictive models, and detail the processes of model construction, validation, and assessment.

#### 2.1.2 Exclusion criteria

① Studies solely focusing on risk factors without comprehensive risk modeling. ② Case series, case reports, randomized controlled trials, and descriptive surveys. ③ Guidelines, expert opinions, reviews, and animal studies. ④ Studies not published in English. ⑤ Studies where the original text was inaccessible or the information was incomplete.

### 2.2 Literature search strategy

**Figure 1:**
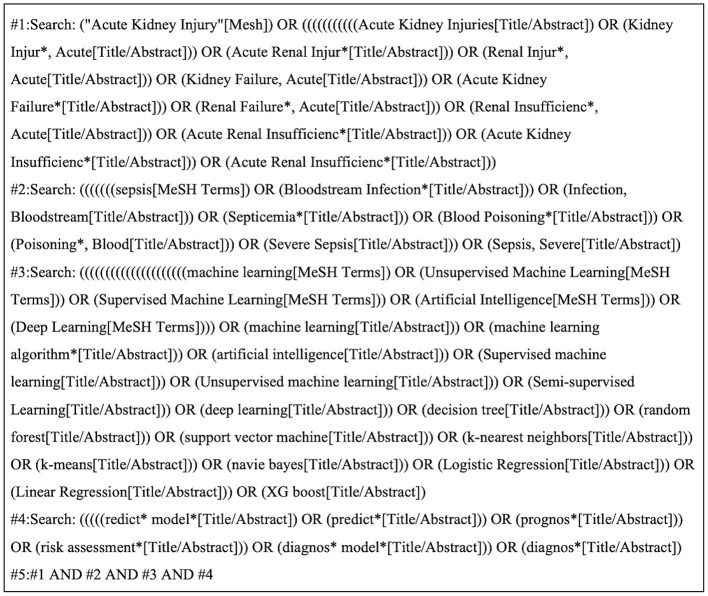
PubMed search strategy.

A systematic search was conducted across four databases: PubMed, Embase, Web of Science, and Cochrane Library. The search utilized a combination of terms including “Acute Kidney Injury/Acute Renal Injury/Acute Kidney Failure,” “Sepsis/Bloodstream Infection,” “Machine Learning/Artificial Intelligence/decision tree/random forest/support vector machine/k-nearest neighbors/k-means/naive bayes/Logistic Regression/Linear Regression/XGboost,” and “Prediction model/risk assessment.” Both subject headings and free-text keywords were employed. The search encompassed records from the inception of each database through March 2025. The search strategy is based on PubMed as an example (Figure 1). The detailed search strategy is provided in the Supplementary material.

### 2.3 Literature screening and data extraction

After completing the literature search, we imported the results into EndNote 21 for management. Two researchers conducted the literature screening by reviewing the titles, abstracts, and additional relevant information of the documents, strictly adhering to the predefined inclusion and exclusion criteria to identify eligible studies. In cases where the two researchers disagreed on the final selection of a study, they consulted a third researcher to reach a consensus. Once a study was confirmed for inclusion, the full article was downloaded and thoroughly read. Data collection followed the Cochrane guidelines and adhered to the Critical Appraisal and Data Extraction Checklist for the Systematic Evaluation of Predictive Models (CHARMS) (17). The extracted data included: publication year, study type, country, data source, patient characteristics, diagnostic criteria for AKI, study endpoints, methods for addressing missing variables, predictor screening, ML algorithms, validation types, calibration metrics, and other relevant details.

### 2.4 Risk of bias and applicability assessment

The risk of bias in the quality of models for included studies was independently assessed by two investigators using the PROBAST-AI assessment tool (18). This tool is an advanced version of PROBAST, specifically designed to assess the risk of bias and clinical applicability of predictive models in healthcare, including both traditional regression and AI/ML models. Introduced to address the swift advancements in AI technologies and the accompanying methodological challenges, PROBAST-AI enhances compatibility with AI/ML technologies. It supports the detailed assessment of ML models, focuses on fairness by incorporating new criteria for evaluating algorithmic bias and data representativeness, and categorizes performance validation. In terms of risk of bias assessment, PROBAST-AI evaluates 18 questions across four domains: participant and data source, predictor, outcome, and statistical analysis. Responses to each question are categorized as “yes/maybe,” “no/could be,” or “unclear.” A domain is considered to have a low risk of bias if all responses are “yes/maybe;” it has a high risk of bias if any response is “no/could be.” If a domain has an “unclear” response to any question while the rest are “yes/maybe,” the risk of bias for that domain is considered unclear. When all domains are rated as having low risk of bias, the overall assessment of the study is “low risk of bias.” Conversely, if at least one domain is rated as having a high risk of bias, the overall assessment is “high risk of bias.” If no domain is rated as high risk but at least one is unclear, the overall assessment is “unclear.” The assessment of applicability, based on the first three domains, follows a similar pattern to the risk of bias assessment, with each domain being rated as “good applicability,” “poor applicability,” or “uncertain applicability.”

## 3 Results

### 3.1 Literature screening process and results

**Figure 2:**
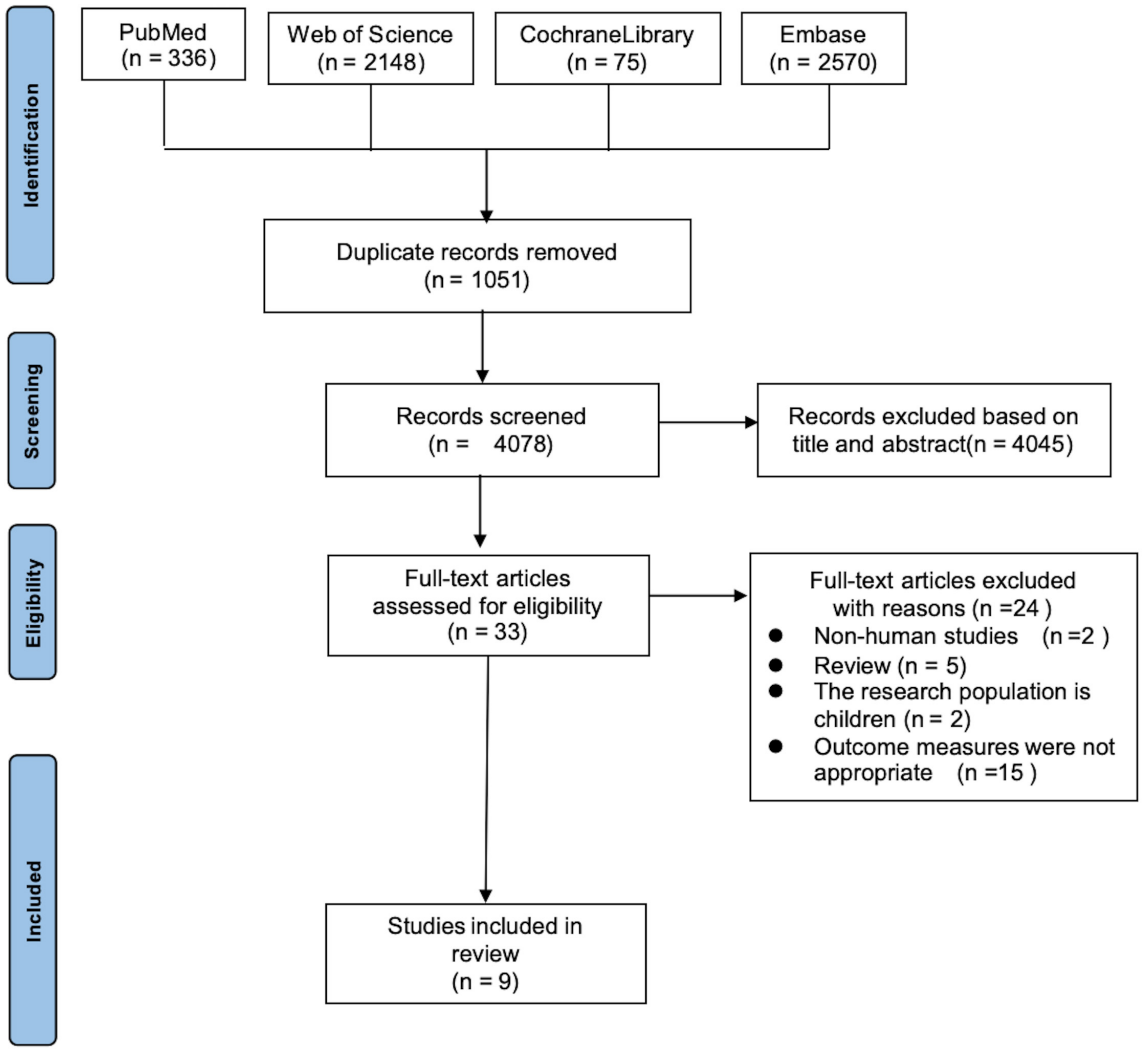
PRISMA study selection flow chart.

After the initial search, 5,129 documents were retrieved. Following the removal of duplicates using EndNote software, 4,078 documents remained. After further screening, 9 documents were ultimately included in the study. The literature screening process is depicted in Figure 2.

### 3.2 Basic characteristics of included literature

Nine studies, published between 2022 and 2024, involved investigators all based in China. One study (19) focused on elderly patients aged 65 years or older, while eight studies (20–27) included adult patients aged 18 years and older. All were retrospective cohort studies. Data were sourced from public databases such as MIMIC-IV, MIMIC-III, eICU, and hospital electronic medical record systems. Five of the studies (19–21, 24, 25) examined in-hospital mortality outcomes, three studies (22, 23, 26) investigated mortality 28 days post-ICU admission, and one study (27) explored 1-year mortality outcomes. For additional details on the included literature, refer to Table 1.

**Table 1:** Basic information on the included literature.

**Author**	**Year**	**Country**	**Disease background**	**Study type**	**Data source**	**AKI diagnostic criteria**	**Predicted outcomes**
Jie Tang (19)	2024	China	ICU sepsis patients over 65 years old	Retrospective cohort study	MIMIC-IV database	2012 KDIGO	Hospitalized mortality rate
Xunliang Li (20)	2023	China	Adult septic patients with AKI within 48 h of ICU admission	Retrospective cohort study	MIMIC-IV database	2012 KDIGO	Hospitalized mortality rate
Hongshan Zhou (21)	2023	China	Patients over 18 years old with sepsis admitted to ICUs	Retrospective cohort study	MIMIC-IV database, Xiangya Hospital of Central South University, Xiangya Third Hospital of Central South University, China	2012 KDIGO	Hospitalized mortality rate
XiaoQin Luo (22)	2022	China	Patients over 18 years of age with sepsis admitted to ICUs	Retrospective cohort study	MIMIC-IV database, eICU database	2012 KDIGO	28-day mortality rate
Jijun Yang (23)	2023	China	Patients over 18 years of age with sepsis admitted to ICUs	Retrospective cohort study	MIMIC-IV database	2012 KDIGO	28-day mortality rate
Tianyun Gao (24)	2024	China	Patients over 18 years of age with sepsis admitted to ICUs	Retrospective cohort study	MIMIC-IV database	2012 KDIGO	Hospitalized mortality rate
Lei Dong (25)	2024	China	Patients aged 18-89 years with sepsis admitted to ICUs	Retrospective cohort study	MIMIC-IV database, MIMIC-III database, Beijing Friendship Hospital ICU	2012 KDIGO	Hospitalized mortality rate
Zhiyan Fan (26)	2023	China	Patients aged 18 years or older with sepsis admitted to ICUs	Retrospective cohort study	MIMIC-IV database, Hangzhou First People's Hospital	2012 KDIGO	28-day mortality rate
Le Li (27)	2024	China	Patients over 18 years of age with sepsis admitted to ICUs	Retrospective cohort study	MIMIC-IV database, MIMIC-III database	2012 KDIGO	1-year mortality rate

### 3.3 Inclusion of literature in predictive model construction

**Figure 3:**
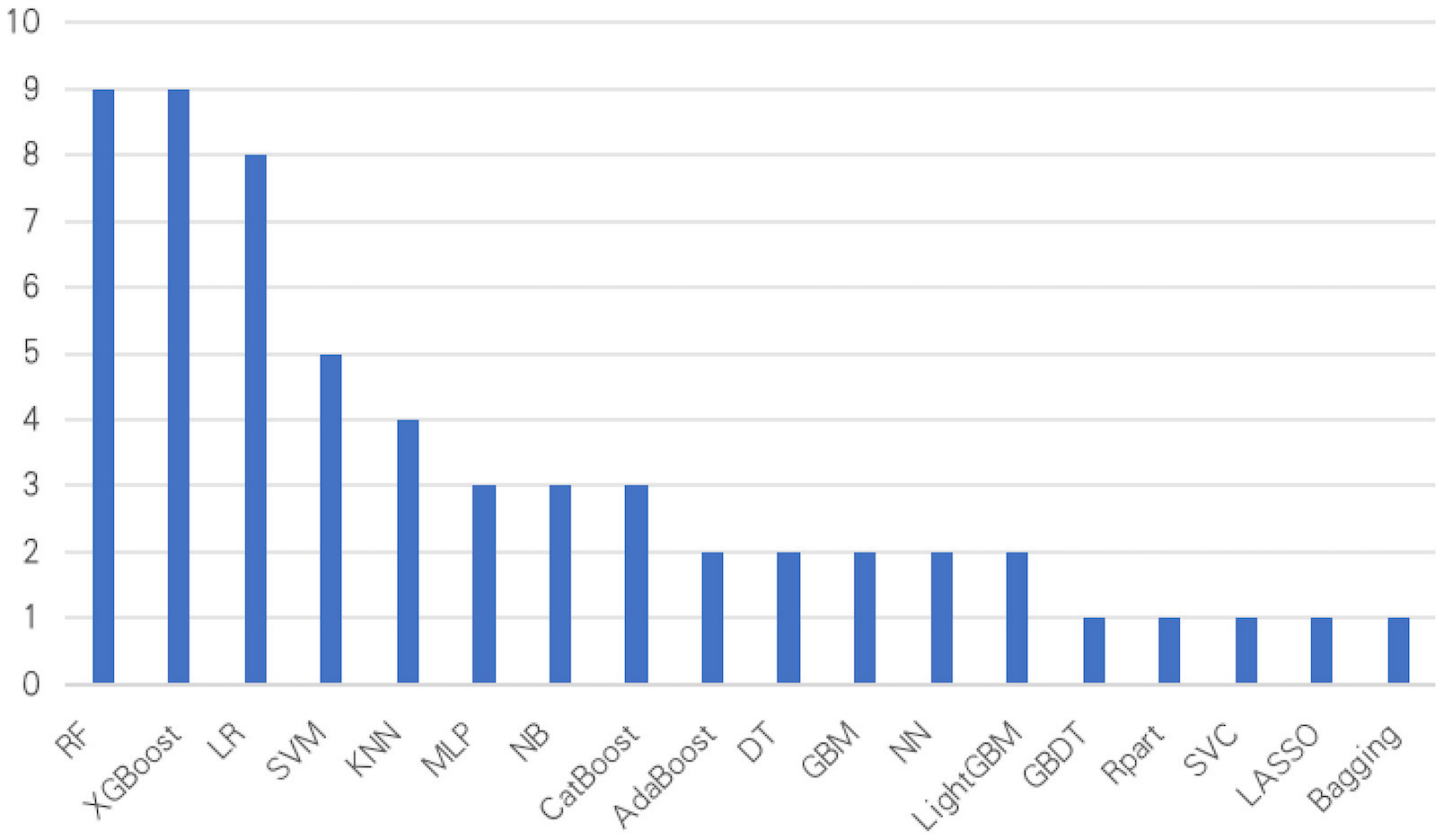
Distribution of ML algorithms.

Among the nine included studies, details of the model construction are presented in Table 2. The training set sample size ranged from 1,999 to 12,923, the internal validation set sample size from 500 to 3,231, and the external validation set sample size from 100 to 3,471. All studies focused on developing and validating predictive models. Of these, five studies (19, 20, 22–24) conducted only internal validation, while the remaining four studies (21, 25–27) conducted external validation. The ML algorithms used are displayed in Figure 3, encompassing 18 types, including: Random Forest (RF), Extreme Gradient Boosting (XGBoost), Logistic Regression (LR), Support Vector Machine (SVM), K-Nearest Neighbors (KNN), Multilayer Perceptron (MLP), Naive Bayes (NB), Adaptive Boosting (AdaBoost), Categorical Boosting (CatBoost), Decision Tree (DT), Gradient Boosting Machine (GBM), Neural Network (NN), Gradient Boosting Decision Tree (GBDT), Light Gradient Boosting Machine (LightGBM), Recursive Partitioning and Regression Trees (Rpart), Support Vector Classifier (SVC), Least Absolute Shrinkage and Selection Operator (LASSO), and Bootstrap Aggregating (Bagging). Among these, RF and XGBoost were the most frequently used (*n* = 9), followed by LR (*n* = 8), SVM (*n* = 5), and KNN (*n* = 4). XGBoost exhibited the best predictive performance in five studies (20, 22, 23, 25, 26), CatBoost in three studies (19, 21, 27), and RF in one study (24). Six studies (19, 23–27) reported deleting missing variable values; three studies (19, 21, 25) used KNN for missing value imputation, four studies (20, 24, 26, 27) used Multiple Imputation by Chained Equations (MICE), one study (22) used XGBoost, and one study (23) used RF. Three studies (19, 21, 26) employed Recursive Feature Elimination (RFE) for predictor selection, two studies (20, 26) used LASSO, one study (22) used XGBoost, one study (23) used the Boruta algorithm, two studies (25, 26) used RF, one study (26) used LR, and one study (27) used SHAP values for factor selection. Among the nine included studies, except for two studies (21, 26) that did not mention calibration metrics, the remaining seven studies (19, 20, 22–25, 27) all utilized calibration curves as calibration metrics. Additionally, two studies (25, 27) employed the Brier score and Kappa coefficient for performance assessment.

**Table 2:** Construction of a risk prediction model for SA-AKI based on ML algorithms.

**Authors**	**Sample size D/I/E**	**Types of Predictive Modeling Studies**	**ML algorithms**	**Optimal algorithm**	**Removal of missing variables**	**Interpolation methods**	**Factor filtering methods**	**Validation methods**	**Model checking methods**	**Calibration metrics**
Jie Tang (19)	5,934/2,942/-	Development and validation of	LR, SVM, GBM, AdaBoost, XGBoost, CatBoost, NB, NN, MLP, KNN, RF	CatBoost	>5%	KNN	RFE	Internal validation	Random sampling	Calibration curve
Xunliang Li (20)	6,503/1,626/-	Development and validation of	LR, SVM, KNN, DT, RF, XGBoost	XGBoost	NA	MICE	LASSO	Internal validation	Random sampling	Calibration curves
Hongshan Zhou (21)	12,923/3,231/132	Developed and validated	KNN, AdaBoost, MLP, SVM, LR, NB, GBDT, RF, LightGBM, XGBoost, CatBoost	CatBoost	NA	KNN	RFE	Internal validation + external validation	Random sampling	NA
Authors	Sample size D/I/E	Types of Predictive Modeling Studies	ML algorithms	Optimal algorithm	Removal of missing variables	Interpolation methods	Factor filtering methods	Validation methods	Model checking methods	Calibration metrics
Xiaoqin Luo (22)	6,066/2,427/-	Developed and validated	XGBoost, RF, SVM	XGBoost	NA	XGBoost	XGBoost	Internal validation	Random sampling	Calibration curves
Jijun Yang (23)	6,411/2,747/-	Development and validation of	LR, RF, GBM, XGBoost	XGBoost	>20%.	RF	Boruta	Internal validation	5-fold cross-validation	Calibration curve
Tianyun Gao (24)	9,756/2,440/-	Developed and validated	KNN, XGBoost, NB, DT, SVM, RF, LR	RF	>25%.	MICE	RF	Internal verification	10-fold cross-validation	Calibration curve
Lei Dong (25)	4,001/1,747/1,829	Developed and validated	LR, Lasso, Rpart, RF, XGBoost, NN	XGBoost	>30%	KNN	LR, Lasso, RF	Internal validation + external validation	Bootstrap sampling	Calibration curves, Brier scores, and kappa coefficients
Zhiyan Fan (26)	1,999/500/100	Developed and validated	RF, SVC, LR, XGBoost, MLP	XGBoost	>30%	MICE	RFE	Internal validation + external validation	Random sampling	NA
Le Li (27)	10,200/2,550/1,658	Developed and validated	LightGBM, XGBoost, CatBoost, RF, LR, Bagging	CatBoost	>30%	MICE	SHAP value	Internal validation + external validation	Random sampling	Calibration curves, Brier scores

### 3.4 Performance of the literature model and prediction factor results

The nine studies encompassed in this investigation consistently reported the Area Under the Curve (AUC) scores for models utilizing the predictive factors outlined in Table 3. Distinct differences in model performance emerged between the training sets and the internal validation sets. For instance, in the study conducted by Tang (19), the RF training set achieved an AUC of 0.99, whereas the AUC for the internal validation set decreased to 0.80, suggesting an overfitting problem. Conversely, the XGBoost model analyzed by Dong exhibited robust performance, with AUC scores of 0.94 in the training set, 0.86 in the internal validation set, and 0.89 in the external validation set, indicating a strong generalization capability. Zhou et al.'s (21) CatBoost model displayed consistent stability across the training set (0.83), internal validation set (0.75), and external validation set (0.75). Regarding external validation, four studies (22, 25–27) provided results, with notable performances from Dong et al.'s (25) XGBoost at 0.89, Fan et al.'s (26) XGBoost at 0.79, and Li et al.'s (27) CatBoost at 0.78, whereas the external validation AUCs for other models predominantly fell below 0.8. The predictive factors incorporated in the final analysis were categorized into six groups: demographic information, vital signs, laboratory test indicators, disease severity, comorbidities, and treatment interventions. These factors included AKI stage, arterial oxygen partial pressure, lactate, urine output, norepinephrine (dose/injection rate), blood urea nitrogen (BUN), invasive mechanical ventilation, base excess, anion gap, age, weight, prevalence of cerebrovascular disease, diabetes, rheumatic disease, paraplegia, liver disease, cancer, heart rate, respiratory rate, body temperature, creatinine, serum chloride, hemoglobin, platelets, white blood cells, international normalized ratio (INR), serum sodium, prothrombin time, SOFA score, SAPS II score, red blood cell distribution width, inspired oxygen fraction, Glasgow Coma Scale (GCS) score, gender, race, hours post-admission, systolic blood pressure, diastolic blood pressure, oxygen saturation, serum total bilirubin, albumin, arterial blood carbon dioxide partial pressure, serum potassium, serum bicarbonate, partial thromboplastin time, mechanical ventilation, use of vasopressors, renal replacement therapy, loop diuretics, eosinophils, monocytes, lymphocyte-to-monocyte ratio, cardiovascular disease, neutrophils, neutrophil-to-lymphocyte ratio, dementia, mean arterial pressure, ROX-heart rate, hemoglobin concentration, ICU length of stay, hypertension, chronic kidney disease, aspartate aminotransferase, shadow value, acute myocardial infarction, congestive heart failure, atrial fibrillation, atrial and atrioventricular pacing, left bundle branch block, ST segment, ventricular tachycardia, BMI, oxygenation index, arterial blood pH.

**Table 3:** Performance of the ML-based predictive model for SA-AKI risk and final included predictors.

**Authors**	**Training set AUC**	**Internal validation set AUC**	**External validation set AUC**	**Predictors ultimately included**
Jie Tang (19)	AdaBoost:0.92 GBM:0.86 KNN:0.82 LR:0.79 MLP:0.81 NB:0.80 NN:0.81 RF:0.99 SVM:0.79 XGBoost:0.84 CatBoost:0.84	AdaBoost:0.75 GBM:0.80 KNN:0.79 LR:0.77 MLP:0.79 NB:0.79 NN:0.79 RF:0.80 SVM:0.76 XGBoost:0.79 CatBoost:0.80	-	10 items: AKI staging, arterial oxygen partial pressure, lactate levels, urine output, norepinephrine dosage, BUN levels, invasive mechanical ventilation, base excess, and anion gap
Xunliang Li (20)	-	LR:0.73 SVM:0.68 KNN:0.60 DT:0.59 RF:0.78 XGBoost:0.79	-	24 items: age, weight, prevalence of cerebrovascular disease, diabetes, rheumatic disease, paraplegia, liver disease, cancer, heart rate, respiratory rate, body temperature, creatinine, serum chloride, hemoglobin, platelets, anion gap, white blood cell count, INR, serum sodium concentration, BUN, prothrombin time, urine output, SOFA score, SAPS II score
Hongshan Zhou (21)	CatBoost:0.83 GBDT:0.82 GBM:0.82 AdaBoost:0.82 RF:0.82 XGBoost:0.81 KNN:0.80 MLP:0.79 LR:0.79 NB:0.76 SVM:0.76	CatBoost:0.75	CatBoost:0.75	15 items: urine output, maximum BUN, norepinephrine infusion rate, maximum anion gap, maximum creatinine, maximum red blood cell distribution width, minimum INR, maximum heart rate, maximum body temperature, maximum respiratory rate, minimum inspired oxygen fraction, minimum creatinine, minimum GCS score, and diagnoses of diabetes and stroke
Xiaoqin Luo (22)	-	XGBoot:0.80 RF:0.80 SVM:0.77	XGBoost:0.75 RF:0.75 SVM:0.72	34 items: age, gender, ethnicity, hours post-admission, systolic blood pressure, diastolic blood pressure, heart rate, respiratory rate, body temperature, oxygen saturation, GCS score, urine output, baseline serum creatinine, hemoglobin, white blood cell count, platelet count, serum total bilirubin, human serum albumin, serum creatinine, BUN, arterial blood pH, arterial blood oxygen partial pressure, arterial blood carbon dioxide partial pressure, serum sodium, serum potassium, serum chloride, serum bicarbonate, lactate, INR, partial thromboplastin time, mechanical ventilation, use of vasopressors, renal replacement therapy, loop diuretics.
Jijun Yang (23)	-	LR:0.85 RF:0.85 GBM:0.87 XGBoost:0.87	-	50 items: minimum shadow value, diabetes without complications, average shadow value, diabetes with complications, acute myocardial infarction, congestive heart failure, gender, ventilation status, acute kidney injury stage, paraplegia, maximum shadow value, eosinophils, respiratory rate, monocytes, albumin, calcium, hemoglobin, lymphocyte-to-monocyte ratio, cancer, cardiovascular disease, neutrophils, neutrophil-to-lymphocyte ratio, white blood cells, liver-related diseases, liver-related diseases, systolic blood pressure, dementia, mean arterial pressure, potassium, heart rate, ROX-Heart rate, diastolic blood pressure, arterial blood carbon dioxide partial pressure, body mass index, oxygenation index, blood oxygen saturation, blood glucose, arterial blood oxygen partial pressure, sodium, bicarbonate, creatinine, acid-base balance, age, chloride, body temperature, lactate, anion gap, solid tumors, BUN, urine output
Tianyun Gao (24)	-	KNN:0.69 XGBoost:0.80 NB:0.76 DT:0.64 SVM:0.72 RF:0.80 LR:0.76	-	11 items: GCS score, AKI grading, SAPS II score, respiratory rate, creatinine level, sodium level, BMI, absolute lymphocyte count, urine output, age, and temperature.
Lei Dong (25)	LR:0.84 Lasso:0.83 Rpart:0.75 RF:0.88 XGBoost:0.94 NN:0.87	LR:0.82 Lasso:0.81 Rpart:0.73 RF:0.80 XGBoost:0.86 NN:0.82	LR:0.75 Lasso:0.73 Rpart:0.60 RF:0.64 XGBoost:0.89 NN:0.74	42 items: unspecified primary hypertension, type 2 diabetes without mention of complications, unspecified congestive heart failure, acute hemorrhagic anemia, acute respiratory failure with hypoxemia, acute respiratory failure, atrial fibrillation, atrial and atrioventricular pacing, left bundle branch block, ST segment, ventricular tachycardia, invasive ventilation, age, maximum blood gas lactate level, maximum blood gas oxygen partial pressure, minimum blood gas carbon dioxide partial pressure, maximum blood gas carbon dioxide partial pressure, minimum blood gas base excess, maximum blood gas base excess, minimum blood gas calcium level, maximum blood gas calcium level, minimum GCS score, maximum heart rate, minimum systolic blood pressure, minimum respiratory rate, maximum respiratory rate, maximum body temperature, minimum blood oxygen saturation, minimum blood glucose, urine output, minimum platelet count, maximum anion gap, maximum BUN, maximum blood calcium, maximum blood chloride, maximum blood creatinine, minimum prothrombin time, maximum prothrombin time, minimum partial thromboplastin time, maximum partial thromboplastin time.
Zhiyan Fan (26)	RF:0.79 SVC:0.76 LR:0.74 XGBoost:0.83 MLP:0.79	-	RF:0.67 SVC:0.69 LR:0.67 XGBoost:0.79 MLP:0.73	40 items: SOFA score, AKI stage III, minimum blood glucose level, minimum white blood cell count, mean oxygen saturation, maximum creatinine level, maximum sodium level, urine output, maximum white blood cell count, minimum lactate level, body weight, mean heart rate, minimum mean arterial pressure, maximum blood glucose level, minimum platelet count, minimum hematocrit, mean arterial pressure, age at admission, minimum potassium level, minimum creatinine level, maximum body temperature, maximum mean arterial pressure, minimum heart rate, minimum respiratory rate, minimum bicarbonate level, maximum hematocrit, maximum INR, maximum platelet count, average respiratory rate, maximum anion gap, minimum BUN level, maximum hemoglobin level, maximum bicarbonate level, minimum body temperature, minimum blood oxygen saturation, maximum BUN level, average body temperature, maximum lactate level, average blood glucose level, maximum heart rate.
Le Li (27)	-	CatBoost: 0.81 LightGBM:0.80 XGBoost:0.79 RF:0.79 LR:0.79 Bagging:0.74	CatBoost: 0.78 LightGBM:0.77 XGBoost:0.75 RF:0.76 LR:0.77 Bagging:0.68	10 items: age, ICU length of stay, GCS score, hypertension, chronic kidney disease, creatinine, BUN, aspartate aminotransferase, hemoglobin, and urine output.

### 3.5 Literature bias risk and applicability assessment results

The assessment results of predictive model studies using PROBAST-AI indicated that nine studies exhibited a high overall bias risk in terms of bias risk assessment. Although these studies were retrospective and utilized data from publicly available large databases with adequate sample sizes and multi-center data, their risk in the areas of participants and data sources remained low. This was due to the clearly defined inclusion and exclusion criteria in the study design, ensuring low bias risk in these areas. In the predictive factor domain, the nine studies had clearly defined predictive factors and utilized various factor screening methods for preprocessing. The predictive factors were based on outcome data and were suitable for use in the intended application of the model. However, some studies (19, 22, 25) included treatment intervention factors such as invasive mechanical ventilation, renal replacement therapy, and vasoactive drug use. Including these factors in the prediction might introduce treatment-related bias, resulting in a high risk of bias in this domain. In the outcome indicator domain, outcomes were clearly defined and determined through hospital records within a clear time window, exhibiting no objective bias. All patients had clear outcome records with no loss to follow-up, leading to a low risk in the outcome indicator domain. In the statistical analysis domain, two studies (19, 20) were assessed to have a high risk of bias due to the use of only single-fold cross-validation during internal validation and the absence of cross-validation. Five studies (19, 20, 22–24) were considered to have a high bias risk due to the lack of external validation. Two studies (21, 26) conducted external data validation but were still deemed to have a high bias risk owing to the small sample size of the external validation data. Conversely, two studies (25, 27) used another large-sample database for external validation, which resulted in a low risk of bias. Additionally, two studies (21, 26) were rated as having a high risk of bias due to the absence of calibration metrics. Regarding overall applicability, the nine studies included populations consistent with the model's target population, utilized common predictive factors that matched the intended use, and predicted endpoint measures that were core indicators of S-AKI patient outcomes. Consequently, all were rated as having good applicability. The comprehensive results of the overall bias risk and applicability assessment for the included literature are detailed in Table 4.

**Table 4:** Bias risk and applicability assessment of an ML algorithm-based predictive model for SA-AKI risk.

**Authors**	**Risk of bias**				**Applicability**			**Overall**	
	**Participants**	**Predictors**	**Outcome**	**Analysis**	**Participants**	**Predictors**	**Outcome**	**Risk of bias**	**Applicability**
Jie Tang (19)	-	-	+	-	-	-	-	-	+
Xunliang Li (20)	-	+	+	-	-	-	-	-	+
Hongshan Zhou (21)	-	+	+	-	-	-	-	-	+
XiaoQin Luo (22)	-	-	+	-	-	-	-	-	+
Jijun Yang (23)	-	+	+	-	-	-	-	-	+
Tianyun Gao (24)	-	+	+	-	-	-	-	-	+
Lei Dong (25)	-	-	+	+	-	-	-	-	+
Zhiyan Fan (26)	-	+	+	+	-	-	-	-	+
Le Li (27)	-	+	+	+	-	-	-	+	+

−, high risk of bias; +, low risk of bias.

## 4 Discussion

### 4.1 Performance of mortality risk prediction models for SA-AKI patients based on ML algorithms

In the nine studies included in this research, the performance of various ML models in prediction tasks showed that the AUC on the training set mostly ranged from 0.75 to 0.99, with RF, XGBoost, and CatBoost performing particularly well. Although the AUC on the internal validation set slightly decreased, it still generally remained above 0.70, indicating good model performance. Among these models, XGBoost, CatBoost, and GBM demonstrated strong stability. Only four studies reported results from external validation, which indicated that XGBoost and CatBoost were the most optimal models. Out of the nine studies reviewed, eight were deemed to have a high risk of bias, primarily due to issues within the domains of predictor variables and statistical analysis. ① Predictor domain: the studies in question incorporated treatment intervention variables, including invasive mechanical ventilation, vasopressor use, and renal replacement therapy. These interventions are correlated with patients' baseline characteristics, which in turn can influence outcomes. If the models fail to sufficiently adjust for these confounding factors, the relationship between treatment interventions and outcomes may be misrepresented, thereby inducing prediction bias. Furthermore, treatment interventions might vary over the course of follow-up—for instance, through discontinuation or alteration of medications. Traditional predictive models often presume that predictor variables remain static from baseline. Neglecting the dynamic nature of treatment changes could lead to underestimations or overestimations of their impact on outcomes. Although most studies referenced the employment of factor selection methods such as LASSO, Boruta, and RFE, they failed to confirm whether feature selection and preprocessing were limited to the training datasets, thereby introducing the risk of data leakage and a consequent high risk of bias. ② Statistical analysis field: the discussed literature primarily reports performance metrics like AUC, sensitivity, and specificity. However, several studies neglect to provide calibration plots or Blair coefficients, which hampers the assessment of the reliability of predicted probabilities. Additionally, multiple studies do not specify the rate of missing data nor clarify whether techniques such as imputation, exclusion, or retention were employed. The inconsistency in handling missing data, or the outright deletion thereof, can significantly increase analytical bias. Furthermore, most studies merely implement simple training/testing splits and fail to document whether all modeling steps were replicated during cross-validation, potentially leading to an overestimation of model performance.

### 4.2 Analysis of risk factors for SA-AKI

This systematic review included nine ML-based models for predicting mortality risk in patients with SA-AKI, which, despite variations in algorithm types and feature selection methods, consistently incorporated variables such as demographic information, vital signs, laboratory tests, disease severity, comorbidities, and interventions. Among these, frequently included high-frequency variables were age, urine output, serum creatinine, BUN, respiratory rate, heart rate, lactate, and SOFA score, highlighting their clinical significance and biological relevance in the pathophysiology of SA-AKI. Notably, advanced age was included as a predictive factor in eight of the nine studies, ranking highest. Previous research has established age as a crucial independent factor affecting the mortality risk in SA-AKI patients, primarily due to diminished physiological reserve, compromised immune function, and reduced organ compensatory capacity. A multicenter retrospective cohort study on elderly SA-AKI patients revealed that these patients were more prone to develop SA-AKI and exhibited significantly higher mortality rates. This study also indicated that age positively correlates with the severity of SA-AKI, making it an essential covariate to control (28). Renal function-related indicators such as serum creatinine, urine output, and BUN have been consistently included across multiple studies, reflecting directly on the severity of AKI and demonstrating stable and independent predictive value. A retrospective analysis using the MIMIC-IV database showed (29) that serum creatinine levels at admission and a 24-h increase in creatinine are independent risk factors for persistent SA-AKI leading to mortality. Additionally, other studies have verified that a reduction in urine output on the first day of admission is significantly associated with increased mortality, nearly doubling the risk (30). A multicenter retrospective analysis by Harazim et al. demonstrated that, after adjusting for key confounding variables, elevated BUN levels at admission were significantly associated with an increased risk of 28-day mortality (31). A study utilizing MIMIC-III data, which included over 12,700 sepsis patients, indicated that BUN levels ≥41.1 mg/dL were significantly associated with an increased 30-day mortality risk (32). Additionally, some studies have modeled dynamic forms of these variables, such as maximum values and 24-h averages, highlighting the importance of capturing trends in changes throughout the disease course. This suggests that future model development should focus more on time-sensitive feature engineering methods (33). Regarding vital signs, respiratory rate and heart rate have consistently been included in multiple studies as non-invasive, easily accessible indicators that can promptly reflect systemic stress, infection progression, and organ hypoperfusion. A study employing causal inference analysis discovered that a sustained high heart rate was significantly associated with decreased hospital and 90-day survival rates in patients with SA-AKI (34). Retrospective studies have also confirmed that a respiratory rate exceeding 20 breaths per minute is an independent predictor of irreversible AKI and mortality risk (35). Although these indicators can be influenced by interventions such as sedation and mechanical ventilation (36), their clinical warning value remains significant. Therefore, models should adequately consider these contextual factors during the modeling process. Lactate, as a metabolic indicator, has been repeatedly included in final models. It serves as a crucial indicator of tissue hypoperfusion and acid-base imbalance and has been widely utilized in clinically predicting mortality risk among critically ill patients. Its inclusion improves the model's ability to capture states of metabolic imbalance (37). Several studies have incorporated composite scoring indices, such as the SOFA score and the GCS score, to evaluate multi-organ dysfunction and neurological status. Although these indices possess strong explanatory power, their reliance on multiple variables may hinder their usefulness in real-time decision support systems (38). Future research should investigate simpler scoring alternatives to enhance the efficiency of model deployment. In summary, these studies have identified a set of stable, interpretable key variables that are crucial in the convergence of predictive factors. Future model development should give priority to these robust variables, focusing on their dynamic characteristics. Additionally, these variables can be integrated with others, such as the lactate-to-albumin ratio (39, 40), the urea nitrogen-to-albumin ratio (41, 42), and combinations of SOFA scores with serum biomarkers (43), for constructing models. It is important to find a balance between model interpretability and performance to improve the model's generalization capability and clinical practicality, thereby facilitating its transferability to ICU clinical applications.

### 4.3 Differences among ML algorithms in SA-AKI mortality prediction models

The included studies employed various ML algorithms to develop mortality prediction models for SA-AKI, potentially contributing to variations in reported performance. This analysis involved a total of 18 algorithms, with RF and XGBoost being the most frequently used. XGBoost, in particular, demonstrated superior performance in five studies. As tree-based algorithms, RF and XGBoost have an inherent capacity to capture non-linear relationships and complex interactions between predictors (44)—attributes that are especially pertinent to SA-AKI, where mortality risk may be influenced by interdependent factors such as the interplay between renal function, inflammatory markers, and organ failure status. As ensemble methods aggregating outputs from multiple decision trees, these algorithms often achieve robust predictive performance in clinical datasets, a strength supported by prior empirical evidence (45). Secondly, LR is the most frequently used linear model. Linear models offer distinct advantages in terms of interpretability: their outputs, such as odds ratios for individual predictors, enable clinicians to quantify how specific variables (46) (e.g., serum creatinine levels, sepsis onset time) contribute to the mortality risk associated with SA-AKI. This feature enhances clinical trust in model outputs. However, this interpretability comes with a significant limitation: linear models assume a linear relationship between predictors and the mortality outcome, which may not adequately capture the complex, non-linear patterns intrinsic to SA-AKI pathophysiology (47). Consequently, these models may exhibit suboptimal predictive performance compared to more flexible algorithmic approaches. SVMs were also employed in select studies, leveraging their strength in handling high-dimensional datasets—datasets that may include multiple laboratory parameters, vital signs, and comorbidity indicators (48). For SA-AKI mortality classification tasks, SVMs can deliver strong performance, but their efficacy is highly dependent on two critical steps: selecting an appropriate kernel function (to transform data into a separable space) and rigorous parameter tuning [e.g., adjusting regularization parameters to balance model complexity and generalizability (49, 50)]. For advancing research in mortality prediction related to SA-AKI, it is imperative to possess a sophisticated comprehension of the strengths and limitations inherent to each algorithm, thereby facilitating informed decisions regarding model selection. This comprehension must be aligned with the specific objectives of each study; for instance, tree-based algorithms or neural networks may be prioritized when the primary goal is to maximize predictive accuracy, whereas linear models may be preferable when interpretability and clinical transparency are of utmost importance. Such deliberate selection of algorithms not only augments the reliability of individual studies but also enhances the comparability and cumulative value of evidence within the field of SA-AKI mortality prediction research.

### 4.4 Implications for future research and practice

Currently, the vast majority of studies are plagued by issues such as the improper handling of missing data and the failure to strictly distinguish between training and testing sets during feature selection and data splitting. These shortcomings can lead to overfitting and an overestimation of model performance. Future research should adhere strictly to guidelines such as TRIPOD-AI and PROBAST-AI to standardize data processing, cross-validation, feature selection, and other processes. This adherence will enhance the transparency and reproducibility of models. This study has identified that the frequently included predictive factors—age, urine output, serum creatinine, BUN, respiratory rate, heart rate, lactate, and SOFA score—are mostly available clinically and are highly relevant pathophysiologically. In contrast, some studies have introduced treatment-related variables, such as CRRT, mechanical ventilation, and vasopressor use. While these variables may improve model performance, they could compromise the model's applicability in prospective deployments. Future research should focus on developing models based on “early-available variables” to facilitate the development of clinical early warning systems. Additionally, attention should be directed toward the model's integrability into clinical workflows. This integration could include interfacing with electronic health record systems, automating the triggering of predictions and risk alerts, and ensuring the comprehensibility and trustworthiness of the model's explanations for clinical healthcare providers. By doing so, the complementary and collaborative use of artificial intelligence and clinical judgment could be promoted effectively.

### 4.5 Limitations of the study

The predominant number of prediction models discussed in this analysis were developed and validated exclusively using the MIMIC database. This database primarily comprises data from a single academic medical center in the United States. Relying on this singular data source introduces inherent ethnic and geographic selection biases during the model development process. The demographic and clinical characteristics documented in MIMIC, which include genetic ancestry, socioeconomic status, healthcare delivery protocols, and disease prevalence patterns, do not mirror the diversity of the global population. This is particularly true for individuals from low- and middle-income countries or those belonging to non-Western ethnic groups. Consequently, the performance and reliability of these models may not be applicable to diverse patient populations. Therefore, any conclusions derived from their application should be interpreted with considerable caution to prevent overgeneralization. A second significant limitation arises from the geographic homogeneity of the studies included in this review. All nine investigations that satisfied the predefined inclusion criteria were conducted exclusively in China. This concentration of studies persists despite the research team's adherence to stringent systematic review protocols. These included comprehensive searches across multiple international databases (e.g., PubMed, Embase, the Cochrane Library, and Web of Science) without regional or language restrictions, and strict compliance with the inclusion and exclusion criteria established a priori to minimize selection bias. The absence of eligible studies from other regions, such as North America, Europe, Africa, or Southeast Asia, markedly limits the external validity of the review's findings. Differences in clinical practice guidelines, healthcare systems, and patient demographics across various global contexts can significantly affect the performance of prediction models in real-world clinical settings. To address these deficiencies and enhance the scientific evidence base for these prediction models, future research should prioritize multi-center, multi-ethnic, and international collaborative studies. Such research endeavors should aim to integrate clinical data from geographically diverse populations and multiple healthcare systems. By doing so, researchers can mitigate the impact of biases associated with single-region or single-population data, refine model parameters to account for global variability in patient characteristics, and ultimately improve the models' generalizability and clinical utility on a global scale. These efforts are not merely advantageous but imperative for ensuring that prediction models facilitate equitable, evidence-based healthcare decision-making across the entire spectrum of global patient populations.

## 5 Conclusion

This systematic review rigorously evaluated nine ML-based predictive models for mortality risk in patients with SA-AKI, highlighting prevalent issues and trends in the realms of model design, variable selection, and performance assessment. Models employing ML algorithms have been shown to predict mortality risk in SA-AKI patients with greater accuracy, thereby demonstrating substantial model applicability. Nevertheless, a high overall risk of bias persists within these predictive models. The predictors ultimately incorporated into the models display a consistent typology. For future research, it is imperative to standardize methodologies concerning data processing, cross-validation, and feature selection to improve the transparency and reproducibility of the models. Additionally, the development of models that utilize “early available variables” warrants exploration. This approach should also account for the dynamic nature of these variables to facilitate dynamic predictions.

## Data Availability

The original contributions presented in the study are included in the article/Supplementary material, further inquiries can be directed to the corresponding authors.

## References

[1] Meyer NJ Prescott HC. Sepsis and septic shock. N Engl J Med. (2024) 391:2133–46. 10.1056/NEJMra2403213 39774315

[2] Rudd KE Johnson SC Agesa KM Shackelford KA Tsoi D Kievlan DR . Global, regional, and national sepsis incidence and mortality, 1990-2017: analysis for the Global Burden of Disease Study. Lancet. (2020) 395:200–11. 10.1016/S0140-6736(19)32989-7 31954465 PMC6970225

[3] Poston JT Koyner JL. Sepsis associated acute kidney injury. BMJ. (2019). 364:k4891. 10.1136/bmj.k4891 30626586 PMC6890472

[4] Liu J Xie H Ye Z Li F Wang L. Rates, predictors, and mortality of sepsis-associated acute kidney injury: a systematic review and meta-analysis. BMC Nephrol. (2020) 21:318. 10.1186/s12882-020-01974-8 32736541 PMC7393862

[5] Stubnya JD Marino L Glaser K Bilotta F. Machine learning-based prediction of acute kidney injury in patients admitted to the ICU with sepsis: a systematic review of clinical evidence. J Crit Intensive Care. (2024) 15:37–43. 10.14744/dcybd.2023.3620

[6] Park H Yang J Chun BC. Assessment of severity scoring systems for predicting mortality in critically ill patients receiving continuous renal replacement therapy. PLoS ONE. (2023) 18:286246. 10.1371/journal.pone.0286246 37228073 PMC10212150

[7] Wang N Wang M Jiang L Du B Zhu B Xi X. The predictive value of the oxford acute severity of illness score for clinical outcomes in patients with acute kidney injury. Ren Fail. (2022) 44:320–8. 10.1080/0886022X.2022.2027247 35168501 PMC8856098

[8] Chen YZ Wang BH Zhao YZ Shao XX Wang MS Ma FH . Metabolomic machine learning predictor for diagnosis and prognosis of gastric cancer. Nat Commun. (2024) 15:1657. 10.1038/s41467-024-46043-y 38395893 PMC10891053

[9] Wang XX Li YF Cao ZX Li YN Cao JY Wang Y . Development and external validation of a machine learning model for cardiac valve calcification early screening in dialysis patients: a multicenter study. Ren Fail. (2025). 47:2491656. 10.1080/0886022X.2025.2491656 40275572 PMC12035951

[10] Meng W Wang A Cong X Xu LR Wang F Shi F. predictive value of machine learning and nomogram models based on brain amyloid SUVR in Alzheimer's disease. Acad Radiol. (2025) 21:S1076-6332(25)00641-5. 10.1016/j.acra.2025.07.003 40695719

[11] Wang Y Luo X Wang J Li W Cui J Li Y. Development and validation of machine learning models for predicting 7-day mortality in critically ill patients with traumatic spinal cord injury: a multicenter retrospective study. Neurocrit Care. (2025) 1–15. 10.1007/s12028-025-02308-y 40563047

[12] Li H Zang Q Li Q Lin Y Duan J Huang J . Development of a machine learning-based predictive model for postoperative delirium in older adult intensive care unit patients: retrospective study. J Med Internet Res. (2025) 27:e67258. 10.2196/67258 40537091 PMC12226778

[13] Zhao W Li X Gao L Ai Z Lu Y Li J . Machine learning-based model for predicting all-cause mortality in severe pneumonia. BMJ Open Respir Res. (2025) 12:e001983. 10.1136/bmjresp-2023-001983 40122535 PMC11934410

[14] Malvasi A Malgieri LE Difonzo T Achiron R Tinelli A Baldini GM . Artificial intelligence dystocia algorithm (AIDA) as a decision support system in transverse fetal head position. J Imaging. (2025) 11:223. 10.3390/jimaging11070223 40710610 PMC12295838

[15] Swanson K Wu E Zhang A Alizadeh AA Zou J. From patterns to patients: advances in clinical machine learning for cancer diagnosis, prognosis, and treatment. Cell. (2023) 186:1772–91. 10.1016/j.cell.2023.01.035 36905928

[16] Chiang DH Jiang Z Tian C Wang CY. Development and validation of a dynamic early warning system with time-varying machine learning models for predicting hemodynamic instability in critical care: a multicohort study. Crit Care. (2025) 29:318. 10.1186/s13054-025-05553-x 40702538 PMC12285179

[17] Moons KGM de Groot JAH Bouwmeester W Vergouwe Y Mallett S Altman DG . Critical appraisal and data extraction for systematic reviews of prediction modelling studies: the charms checklist. PLoS Med. (2014) 11:e1001744. 10.1371/journal.pmed.1001744 25314315 PMC4196729

[18] Moons KGM Damen JAA Kaul T Hooft L Andaur Navarro C Dhiman P . PROBAST+AI: an updated quality, risk of bias, and applicability assessment tool for prediction models using regression or artificial intelligence methods. BMJ. (2025) 388:e082505. 10.1136/bmj-2024-082505 40127903 PMC11931409

[19] Tang J Huang J He X Zou S Gong L Yuan Q . The prediction of in-hospital mortality in elderly patients with sepsis-associated acute kidney injury utilizing machine learning models. Heliyon. (2024) 10:e26570. 10.1016/j.heliyon.2024.e26570 38420451 PMC10901004

[20] Li X Wu R Zhao W Shi R Zhu Y Wang Z . Machine learning algorithm to predict mortality in critically ill patients with sepsis-associated acute kidney injury. Sci Rep. (2023) 13:5223. 10.1038/s41598-023-32160-z 36997585 PMC10063657

[21] Zhou H Liu L Zhao Q Jin X Peng Z Wang W . Machine learning for the prediction of all-cause mortality in patients with sepsis-associated acute kidney injury during hospitalization. Front Immunol. (2023) 14:1140755. 10.3389/fimmu.2023.1140755 37077912 PMC10106833

[22] Luo X-Q Yan P Duan S-B Kang Y-X Deng Y-H Liu Q . Development and validation of machine learning models for real-time mortality prediction in critically ill patients with sepsis-associated acute kidney injury. Front Med. (2022) 9:853102. 10.3389/fmed.2022.853102 35783603 PMC9240603

[23] Yang J Peng H Luo Y Zhu T Xie L. Explainable ensemble machine learning model for prediction of 28-day mortality risk in patients with sepsis-associated acute kidney injury. Front Med. (2023) 10:1165129. 10.3389/fmed.2023.1165129 37275353 PMC10232880

[24] Gao T Nong Z Luo Y Mo M Chen Z Yang Z . Machine learning-based prediction of in-hospital mortality for critically ill patients with sepsis-associated acute kidney injury. Ren Fail. (2024) 46:2316267. 10.1080/0886022X.2024.2316267 38369749 PMC10878338

[25] Dong L Liu P Qi Z Lin J Duan M. Development and validation of a machine-learning model for predicting the risk of death in sepsis patients with acute kidney injury. Heliyon. (2024) 10:e29985. 10.1016/j.heliyon.2024.e29985 38699001 PMC11064448

[26] Fan Z Jiang J Xiao C Chen Y Xia Q Wang J . Construction and validation of prognostic models in critically Ill patients with sepsis-associated acute kidney injury: interpretable machine learning approach. J Transl Med. (2023) 21:406. 10.1186/s12967-023-04205-4 37349774 PMC10286378

[27] Li L Guan J Peng X Zhou L Zhang Z Ding L . Machine learning for the prediction of 1-year mortality in patients with sepsis-associated acute kidney injury. BMC Med Inform Decis Mak. (2024) 24:208. 10.1186/s12911-024-02583-3 39054463 PMC11271185

[28] Takeuchi T Flannery AH Liu LJ Ghazi L Cama-Olivares A Fushimi K . Epidemiology of sepsis-associated acute kidney injury in the ICU with contemporary consensus definitions. Critical Care. (2025) 29:128. 10.1186/s13054-025-05351-5 40114218 PMC11924826

[29] Xia W Yi F Wang Q. Mortality and differential predictive factors of transient and persistent sepsis-associated acute kidney injury. Clin Nephrol. (2023) 99:119–27. 10.5414/CN110926 36546763

[30] Yamamoto R Yamakawa K Yoshizawa J Kaito D Umemura Y Homma K . Urine output and development of acute kidney injury in sepsis: a multicenter observational study. J Intensive Care Med. (2025) 40:191–9. 10.1177/08850666241268390 39094594

[31] Harazim M Tan K Nalos M Matejovic M. Blood urea nitrogen - independent marker of mortality in sepsis. Biomed Pap Med Fac Univ Palacky Olomouc Czech Repub. (2023) 167:24–9. 10.5507/bp.2022.015 35373784

[32] Li X Zheng R Zhang T Zeng Z Li H Liu J. Association between blood urea nitrogen and 30-day mortality in patients with sepsis: a retrospective analysis. Ann Palliat Med. (2021) 10:11653–63. 10.21037/apm-21-2937 34872290

[33] Zheng Z Luo J Zhu Y Du L Lan L Zhou X . Development and validation of a dynamic real-time risk prediction model for intensive care units patients based on longitudinal irregular data: multicenter retrospective study. J Med Internet Res. (2025) 27:e69293. 10.2196/69293 40266658 PMC12059492

[34] DengF ZhuC CaoY ZhaoS. Impact of prolonged elevated heart rate on sepsis-associated acute kidney injury patients: a causal inference and prediction study. Kidney Res Clin Pract. (2025) 24:206. 10.23876/j.krcp.24.20640211868

[35] Luo X Yan P Zhang N Wang M Deng Y Wu T . Early recovery status and outcomes after sepsis-associated acute kidney injury in critically ill patients. Zhong Nan Da Xue Xue Bao Yi Xue Ban. (2022) 47:535–45. 10.11817/j.issn.1672-7347.2022.210368 35753723 PMC10929915

[36] Quickfall D Sklar MC Tomlinson G Orchanian-Cheff A Goligher EC. The influence of drugs used for sedation during mechanical ventilation on respiratory pattern during unassisted breathing and assisted mechanical ventilation: a physiological systematic review and meta-analysis. EClinicalMedicine. (2024) 68:102417. 10.1016/j.eclinm.2023.102417 38235422 PMC10789641

[37] Casserly B Phillips GS Schorr C Dellinger RP Townsend SR Osborn TM . Lactate measurements in sepsis-induced tissue hypoperfusion: results from the surviving sepsis campaign database. Crit Care Med. (2015) 43:567–73. 10.1097/CCM.0000000000000742 25479113

[38] Liu Z Meng Z Li Y Zhao J Wu S Gou S . Prognostic accuracy of the serum lactate level, the SOFA score and the qSOFA score for mortality among adults with Sepsis. Scand J Trauma Resusc Emerg Med. (2019) 27:51. 10.1186/s13049-019-0609-3 31039813 PMC6492372

[39] Ao T Huang Y Zhen P Hu M. Association between the lactate-to-albumin ratio and sepsis-associated acute kidney injury: a cross-sectional study. Eur J Med Res. (2025) 30:518. 10.1186/s40001-025-02760-8 40551267 PMC12186447

[40] Wang Y Yu H. Association between lactate to albumin ratio and mortality among sepsis associated acute kidney injury patients. BMC Infect Dis. (2025) 25:414. 10.1186/s12879-025-10838-1 40140783 PMC11948962

[41] Ye C Zhu C Hu S Mei Y Yang T. A study on the factors influencing mortality risk in sepsis-induced acute kidney injury based on analysis of the MIMIC database. Clin Exp Med. (2025) 25:192. 10.1007/s10238-025-01681-4 40481893 PMC12145314

[42] Han K Tao Y Wang J Lu J. Prognostic value of blood urea nitrogen to albumin ratio in septic patients with acute kidney injury-a retrospective study based on MIMIC database. Front Med. (2025) 12:1510919. 10.3389/fmed.2025.1510919 40400629 PMC12092353

[43] Lee CW Kou HW Chou HS Chou HH Huang SF Chang CH . A combination of SOFA score and biomarkers gives a better prediction of septic AKI and in-hospital mortality in critically ill surgical patients: a pilot study. World J Emerg Surg. (2018) 13:41. 10.1186/s13017-018-0202-5 30214469 PMC6131912

[44] Meijerink LM Schuit E Moons KGM Leeuwenberg AM. Plug-and-play use of tree-based methods: consequences for clinical prediction modeling. J Clin Epidemiol. (2025) 184:111834. 10.1016/j.jclinepi.2025.111834 40398688

[45] Dragosloveanu S Vulpe DE Andrei CA Nedelea DG Garofil ND Anghel C . Predicting periprosthetic joint Infection: evaluating supervised machine learning models for clinical application. J Orthop Translat. (2025) 54:51–64. 10.1016/j.jot.2025.06.016 40703570 PMC12284488

[46] Franchi De' Cavalieri M Filogna S Martini G Beani E Maselli M Cianchetti M . Wearable accelerometers for measuring and monitoring the motor behaviour of infants with brain damage during CareToy-Revised training. J Neuroeng Rehabil. (2023) 20:62. 10.1186/s12984-023-01182-z 37149595 PMC10164332

[47] Rehman AU Neyra JA Chen J Ghazi L. Machine learning models for acute kidney injury prediction and management: a scoping review of externally validated studies. Crit Rev Clin Lab Sci. (2025) 62:454–76. 10.1080/10408363.2025.2497843 40322979

[48] Zhang Z Chen L Liu H Sun Y Shui P Gao J . Gene signature for the prediction of the trajectories of sepsis-induced acute kidney injury. Crit Care. (2022) 26:398. 10.1186/s13054-022-04234-3 36544199 PMC9773539

[49] Liu L Chu M Gong R Zhang L. An improved nonparallel support vector machine. IEEE Trans Neural Netw Learn Syst. (2021) 32:5129–43. 10.1109/TNNLS.2020.3027062 33055038

[50] Bhatia AS Saggi MK Kais S. Quantum machine learning predicting ADME-Tox properties in drug discovery. J Chem Inf Model. (2023) 63:6476–86. 10.1021/acs.jcim.3c01079 37603536

